# Tissue-Specific Split sfGFP System for Streamlined Expression of GFP Tagged Proteins in the *Caenorhabditis elegans* Germline

**DOI:** 10.1534/g3.119.400162

**Published:** 2019-04-16

**Authors:** Adam Hefel, Sarit Smolikove

**Affiliations:** Department of Biology, University of Iowa, Iowa City, IA 52240

**Keywords:** AKIR-1, CRISPR, germline, GFP1-10, GFP11, meiosis, RPA-1, sfGFP, split GFP, SYP-4

## Abstract

Identifying protein localization is a useful tool in analyzing protein function. Using GFP-fusion tags, researchers can study the function of endogenous proteins in living tissue. However, these tags are considerably large, making them difficult to insert, and they can potentially affect the normal function of these proteins. To improve on these drawbacks, we have adopted the split sfGFP system for studying the localization of proteins in the *Caenorhabditis elegans* germline. This system divides the “super folder” GFP into 2 fragments, allowing researchers to use CRISPR/Cas9 to tag proteins more easily with the smaller subunit, while constitutively expressing the larger subunit from another locus. These two parts are able to stably interact, producing a functional GFP when both fragments are in the same cellular compartment. Our data demonstrate that the split sfGFP system can be adapted for use in *C. elegans* to tag endogenous proteins with relative ease. Strains containing the tags are homozygous viable and fertile. These small subunit tags produce fluorescent signals that matched the localization patterns of the wild-type protein in the gonad. Thus, our study shows that this approach could be used for tissue-specific GFP expression from an endogenous locus.

An informative way to examine the function of a protein is to study the localization of that protein in the cell. The Green Fluorescent Protein (GFP) tag allows investigators to visualize protein localization in living as well as fixed tissues (Chalfie *et al.* 1994). GFP and its derivatives (*e.g.*, RFP, YFP) have become common in genetic research and are frequently introduced as a fusion protein (Wiedenmann *et al.* 2009). Sequences encoding GFP are integrated into the genome or placed outside the genome (*e.g.*, on a plasmid) in frame with a gene of interest. Through the use of CRISPR/Cas9, sequences encoding GFP can be integrated to tag proteins expressed from the normal locus. In most systems, including *C. elegans*, the efficiency of HR is inversely dependent on the size of the insertion, making the insertion of GFP tags relatively challenging (Paix *et al.* 2014; 2017). Due to its large size, a GFP fusion can lead to protein folding issues or perturb protein-protein interactions which can lead to the alteration or disruption of the function of the tagged protein (Snapp 2005). Another limitation of this technology is that GFP fusions illuminate the proteins of interest in all tissue of an organism that express the target protein, which can be problematic when attempting to study the role of a protein in a specific tissue.

One way to generate GFP tagged proteins is through small epitope tag systems such as split-GFP (Ghosh *et al.* 2000). In these systems the sequence encoding for GFP is split into two fragments, and expressed independently. These two fragments are not fluorescent unless they are able to assemble and reconstitute the functional GFP. These split-GFP systems also have the advantage of creating tissue specific reporter tags. The split “super-folder” GFP (split sfGFP) system allows protein folding of the two GFP fragments without the need for assistance by other protein-protein interaction (Ghosh *et al.* 2000; Cabantous *et al.* 2005; Kamiyama *et al.* 2016). sfGFP is different from traditional GFP in that it contains altered residues that promote its stable folding, which were molecularly evolved using DNA shuffling. In the split sfGFP system, the remarkably stable sfGFP is broken into 2 parts, a large subunit containing beta strands 1-10, and a small subunit containing only 1 beta strand. Only when these 2 parts bind each other is fluorescence detected. To regulate tissue-specific fluorescence of target proteins, the large subunit (sfGFP1-10) is constitutively expressed under the regulation of a temporally and/or spatially limited promoter. The large subunit interacts with the smaller subunit (sfGFP11), which can be inserted as a small fusion tag on the protein of interest, forming a functional GFP. This approach was used to tag ribosomes of *C. elegans* with sfGFP11 to visualize their dynamics in neurons (Noma *et al.* 2017). However, this was performed by transgenic integration of both parts of the system, outside the endogenous locus, making it unsuitable for adaptation of CRISPR/Cas9 technology, while potentially adding confounding variables to studying protein function (see discussion). An additional advantage of the split sfGFP system is that the sfGFP11 tags can be tandemly linked, allowing multiple sfGFP1-10 molecules to interact with the protein of interest, thereby improving the fluorescent signal of the tagged protein (Kamiyama *et al.* 2016). This tandem GFP11 repeat approach has not been tested before in *C. elegans*.

In order to study the roles of proteins in the *C. elegans* germline while avoiding the drawbacks of full-length sfGFP tag integration, we have created a streamlined system which uses CRISPR/Cas9 to tag proteins with a split sfGFP. Using MosSCI, we have created a strain of *C. elegans* with constitutive expression of sfGFP1-10 in the germline, allowing for assessment of the dynamics and function of sfGFP tagged proteins of interest in meiosis. The sfGFP1-10 construct used in these experiments utilizes the *pie-1* 5′ UTR and promoter and the *him-3* 3′UTR to restrict expression to the germline. CRISPR/Cas9 was used to insert the sfGFP11 tag at the N-terminus of three tested proteins (AKIR-1, RPA-1, and SYP-4). Using live imaging we were able to detect germline fluorescence of all of the tagged proteins in the *C. elegans* germline. None of these tags appear to have a large effect on the function of these proteins, as assayed by our localization studies and fertility of the strains generated. We also show that adding 3 tandem *gfp11* tags to AKIR-1 mildly improved the fluorescent signal.

## Materials and Methods

### Worm strains and growth conditions

*Caenorhabditis elegans* worms were maintained at 20° on nematode growth media (NGM) plates seeded with *OP50* *Escherichia coli*. When germline silencing was observed, strains were kept at 25° for at least 2 generations before returning to growth at 20°. Strains used for experiments include *N2* (Bristol), and contained the following alleles in the *N2* genetic background:

EG6699 ttTi5605 II; *unc-119(ed3)* III; oxEx1578 [eft-3p::GFP + Cbr-unc-119]
SSM471 *iowSi8*[*pie-1p*::*gfp1-10*::*him-3* 3UTR + Cbr-unc119(+)] II; *unc-119(ed3) III*;
SSM476 *rpa-1*(*iow92*[OLLAS::rpa-1])I
SSM473 *iowSi8*[*pie-1p*::*gfp1-10*::*him-3* 3UTR + Cbr-unc119(+)] II *rpa-1(iow89[gfp11*::*rpa-1])*II; *unc-119*(*ed3*) III;
SSM345 *akir-1*(*iow37*[3xFLAG::akir-1]) 1 I/*hT2* [*bli-4*(*e937*) let-?(*q782*) *qIs48*] (I;III).
SSM472 *akir-1*(*iow88*[gfp11::akir-1])I; *iowSi8*[pie-1p::gfp1-10::him-3 3UTR + Cbr-unc119(+)] II; *unc-119*(*ed3*) III;
SSM474 *syp-4*(*iow90*[gfp11::syp-4])I; *iowSi8*[pie-1p::gfp1-10::him-3 3UTR + Cbr-unc119(+)] II; *unc-119*(*ed3*) III;
*IG1654* wt *frSi12*[pNP157(akir-1p::AKIR-1::GFP)] II

### Plasmid cloning

The plasmid used for generating sfGFP1-10 worms (pSSM334) was cloned using NEBuilder HIFI cloning kit with PCR Primers listed below. For the MosSCI injections, pCFJ150 (Jorgensen laboratory, Plasmid #19329 Addgene) was used as the backbone for cloning for single copy insertion of *gfp1-10*. The *pie-1* promoter and 5′UTR was derived from pCG142 (Seydoux laboratory, Plasmid #17246 Addgene) using the primers 5′- TGTTTGCTCGGCAATC-3′ and 5′- GAAAAGTTGTAGGATCTGGAAG-3′. The *him-3* 3′-UTR was derived from genomic DNA using the primers 5′-ACTATCTCCTCCGAAACTTTCCTGAAATAATAGTCGAAAAGTTTTCACTCATGT-3′ and 5′-CCATGATTACGCCAAGCTCAGAGATTTTGATTTATCTGAACTGGATTTGAATGTT-3′. The plasmid containing sfGFP1-10 was generated using a gBlock from Integrated DNA Technologies (IDT), and primers 5′- TGTTTGCTCGGCAATCG-3′ and 5′-TAATAGTCGAAAAGTTTTCACTCATG-3′. The HIFI reaction mix was allowed 1 hr of incubation due to the large size of the desired plasmid. Sequencing revealed that the sequences were cloned as expected without any modification, except a silent mutation in position 267 of the GFP1-10 coding sequence (CCA to CCG).

### MosSCI

MosSCI injections were performed using the following plasmids; pCFJ601(50 ng/µl), pMA122(10 ng/µl), pGH8(10 ng/µl), pCFJ90(2.5 ng/µl), pCFJ104 (5 ng/µl), and 50 ng/µl of the pCFJ150 plasmid containing our GFP1-10 construct. Injection and maintenance of MosSCI injection worms was adapted from (Frøkjaer-Jensen *et al.* 2008), with 3 injected worms rescued to a plate and incubated at 25° for 1 week before a 34° 2-hour heat shock. Wild-type moving worms with no negative selection markers were isolated and insertion was confirmed with PCR using the primers 5′-TCTGGCTCTGCTTCTTCGTT-3′ and 5′-CAATTCATCCCGGTTTCTGT-3′.

### CRISPR/Cas9 injections

CRISPR/Cas9 was used to create the following strains: *rpa-1*(*iow92*[ollas::rpa-1])I, *iow88*[gfp11::akir-1]; *iow89*[gfp11::rpa-1]; *iow90*[gfp11::syp-4]; *iow91*[3Xgfp11::akir-1]. All strains containing gfp11 were injected into the *iowSi8*[pie-1::gfp1-10::him-3]; *ttTi5605* II; *unc-119*(ed3) III background. Injection of young adult worms was performed on 3% agarose pads, afterward collected on a single NGM plate, and isolated to individual plates the morning following the injection. Plates were screened for the rol or dpy phenotypes created by dpy-10 point mutation introduced by co-CRISPR marker, adopted from (Paix *et al.* 2016). Wild-type F1s were isolated to individual plates for insertion screening by PCR and sequencing. Ultramer oligonucleotides, tracrRNA, and crRNAs were obtained from IDT and mixed in the following concentrations: 14.35 µM Cas9-NLS (Berkeley MacroLab), 17.6 µM tracrRNA (IDT), 1.5 µM dpy10 crRNA (IDT), 5 µM dpy10 ssODN (IDT), 16.2 µM of target crRNA (IDT), and 6 µM of target ssODN (IDT) (see Table 1).

**Table 1:** Sequences used for CRISPR/Cas9, MosSCI, cloning and diagnosis. **Table of the DNA/RNA sequences used for construction of the strains in this manuscript**

Sequence name	Sequence
*ollas*::*rpa-1* ssODN	Ttccccaatttttatgtatctgtttcagatagtgaaagatgtccggattcgccaacgagctcggaccacgtctcatgggaaaggcggcaattcacatcaatcacgatgtcttcaataa
*gfp11*::*rpa-1* ssODN	ttccccaatttttatgtatctgtttcagatagtgaaagatgCGTGACCACATGGTCCTCCACGAGTACGTCAACGCCGCCGGAATCACCGGTGGCGGCAAATTCgcggcaattcacatcaatcacgatgtcttcaataa
*gfp11*::*akir-1* ssODN	ttacttctcgtaaccacaaattatttctttcagaagtaaaatgCGTGACCACATGGTCCTCCACGAGTACGTCAACGCCGCCGGAATCACCGGTGGCGGCAAATTCGCTTGCGGACTCGCACTGAAAAGACCTCTCCAACATGAGTACGAGTCTTTTTTAACTGATGAGACATACAACGGAGAAGCAAAGCGAGCC
*gfp11*::*syp-4* ssODN	gttcggtacggtaacctcatttttcatcaaaattttttatttcaaggcgaaataatgCGTGACCACATGGTCCTCCACGAGTACGTCAACGCCGCCGGAATCACCGGTGGCGGCAAATTCtcgtttccgacgTtacaagtGAgAccaaatgagaaaaatccaaaagttctgcgatgcc
3x*gfp11* ssODN	ATGCGTGACCACATGGTCCTCCACGAGTACGTCAACGCAGCCGGAATCACCGGTGGCGGCAAATTCCGTGACCACATGGTCCTCCACGAGTACGTAAACGCAGCCGGAATCACCGGTGGCGGCAAATTCCGTGACCACATGGTCCTCCACGAGTACGTAAACGCCGCCGGAATCACCGGTGGCGGCAAATTC
*rpa-1 crRNA*	UUUCAGAUAGUGAAAGAUGG
*akir-1 crRNA*	GAUUCAUACUCGUGUUGCAG
*syp-4 crRNA*	UUUGGACGUACUUGUAGCGU
*gfp11 crRNA*	CACGAGUACGUCAACGCCGC
*gfp1-10* g-block with introns	ATGAGTAAAGGAGAAGAATTGTTCACTGGAGTTGTCCCAATCCTCGTCGAGCTCGACGGAGACGTCAACGGACACAAGTTCTCCGTCAGAGGAGAGGGAGAGGGAGACGCCACCATCGGAAAGCTCACCCTCAAGTTCATCTGCACCACCGGAAAGCTCCCAGTCCCATGGCCAACCCTCGTCACCACCTTGACATACGGAGTCCAATGCTTCTCCCGTTACCCAGACCACATGAAGCGTCACGACTTCTTCAAGTCCGCCATGCCAGAGGGATACGTCCAAGAGCGTACCATCTCATTCAAGGTAAGTTTAAACATATATATACTAACTACTGATTATTTAAATTTTCAGGACGACGGAAAATACAAGACCCGTGCCGTGGTCAAGTTCGAGGGAGACACCCTCGTCAACCGTATCGAGCTCAAGGTAAGTTTAAACAGTTCGGTACTAACTAACCATACATATTTAAATTTTCAGGGAACTGACTTCAAGGAGGACGGAAACATCCTCGGACACAAGCTCGAGTACAACTACAACTCCCACAACGTCTACATCACAGCCGACAAGCAAAAGAACGGAATCAAGGCCAACTTCACTATCCGTCACAACATCGAGGACGGATCCGTCCAACTCGCCGACCACTACCAACAAAACACCCCAATCGGAGACGGACCAGTCCTCCTCCCAGACAACCACTACCTCTCCACCCAAACCGTCCTCTCCAAGGACCCAAACGAGAAG
gfp1-10 F	CAAGAGCGTACCATCTCATTCAAG
gfp1-10 R	GTTGTGGGAGTTGTAGTTGTACTC

### Antibody fixation and staining

Gonads were extruded using razor dissection of 10-20 worms in M9 on a coverslip. The coverslip was immediately transferred to a positively charged slide and frozen on dry ice. Preparation of sfGFP worms was performed such that slides were kept in the dark for as long as possible. Fixation was performed with a dip in methanol for 1 min, and a 20 min fix in 4% paraformaldehyde (Alpha Aeser) made from 37% stock. After a 10 min wash in 1XPBST, slides were incubated in the dark with a 4′,6-diamidino-2-phenylindole (DAPI, 1:10,000 of 5mg/ml stock in 1XPBST), followed by a final wash in 1XPBST. Slides were sealed with VECTASHIELD (Vector Laboratories) and stored at 4°.

Preparation of slides for immunofluorescent staining was performed as above, except following fixation slides were stained with antibodies and then DAPI staining was performed. For antibody staining, slides were washed in 1XPBST, incubated with 0.5%BSA in 1XPBST for 1-2 hr after which they were incubated with primary antibody over night at RT. For secondary antibody, slides were washed in 1XPBST three times, incubated with secondary antibody for 2 hr at room temperature in the dark, and followed by 1 wash in 1XPBST and DAPI staining as above. Primary antibodies used were rabbit anti-*SYP-4* (1:500), mouse anti-FLAG (1:500; Sigma) and rabbit anti-OLLAS (1:1000; Genscript #A01658). The secondary antibodies that were used include donkey anti-rabbit Alexa Fluor 488 (1:500; Thermo) and donkey anti-mouse Alexa Fluor 488 (1:500, Thermo).

### Intensity measures

Using FIJI and 16-bit non-deconvolved images, intensity measures were gathered by drawing a circle around a single diakinesis -1 oocyte (the oocyte closes to the spermatheca) from a single slice approximately in the middle of the nucleus. Each image was acquired with a 1 sec FITC channel exposure to maintain consistency. Background was recorded at 5 positions inside the cytoplasmic space and the average was subtracted from each intensity recording. Mann-whitney *U*-test (Graphpad Prism 7 software) was used to compare intensities of GFP.

### Live imaging

For live images, worms were placed on a slide with a 14% agarose in M9 pad and covered with 5ul of a 1:2 mixture of Polybead 0.1-μm polystyrene beads (#00876; Polysciences) in M9. The worms were covered with a coverslip and imaged. All images were taken using the DeltaVision wide-field fluorescence microscope (GE lifesciences) with 100×/1.4 NA oil Olympus objective. Images were deconvolved with softWoRx software (Applied Precision) unless otherwise noted.

### Ethanol fixation

Worms for whole-worm imaging were placed on an uncharged slide (Surgipath Leica) with a drop of M9. The majority of M9 was removed using Whatman filter paper, and 95% ethanol (Millipore) was added to the worms before the worms were allowed to dry. After the ethanol evaporated, 9µl of Vectashield with DAPI was added to the slide, and a #1.5 coverslip was placed on top, before sealing with acrylic nail polish.

### Whole worm imaging

Images were taken on a Leica DMRBE microscope using a 10X/0.30 PL FLUOTAR objective. A QIClick (QIMAGING) camera captured images using Q-Capture software. Scale bars in the whole worm images represent 50µm.

### RT-PCR

For RNA extraction, worms were rinsed from NGM plates using M9, and transferred to a mini-centrifuge tube. Worms were pelleted at 2000x g for 30 sec, and rinsed with M9 2 times, and then pelleted to remove all M9 before the addition of 200µl Triazol. The samples were frozen at -80° for at least one hour, before being thawed and vortexed for 15 sec every 10 min for 1 hr. Samples were spun at 15000x g for 10 min at 4°, followed by addition of 40µl chloroform, vortexing, and incubation on ice for 10 min. Samples were pelleted at 15000x g for 10 min, and 200µl of supernatant was added to a new tube containing 100µl of isopropanol and 1µl of glycogen (20mg/ml). Samples were vortexed and incubated on ice for 10 min, then RNA was pelleted at 15000x g for 10 min at 4°. Pellet was washed with 200µl of 70% ethanol, and then air dried before addition of 45µl of H_2_O. RNA was resuspended and 22µl was transferred to a tube containing 25µl of 2X reaction buffer and 1µl of RT/*taq* mix from Invitrogen’s Superscript III One-step RT-PCR kit. This was split into 2 separate tubes containing 0.5µl of each primer (10mM), before being amplified using conditions described in the kit manual.

### Viability scoring

L4’s were singled onto NGM plates containing a small (1 cm^3^) *OP50* *E. coli* lawn. For 4 days, P0s were transferred to new plates every 12 hr. Eggs were immediately counted, and allowed to hatch and develop into adults before counting adults and 4 days later.

### Data availability

Strains are available upon request. The authors state that all data necessary for confirming the conclusions presented in the article are represented fully within the article.

## Results

### A split sfGFP system for streamlined GFP tagging of proteins in the germline

**Figure 1:**
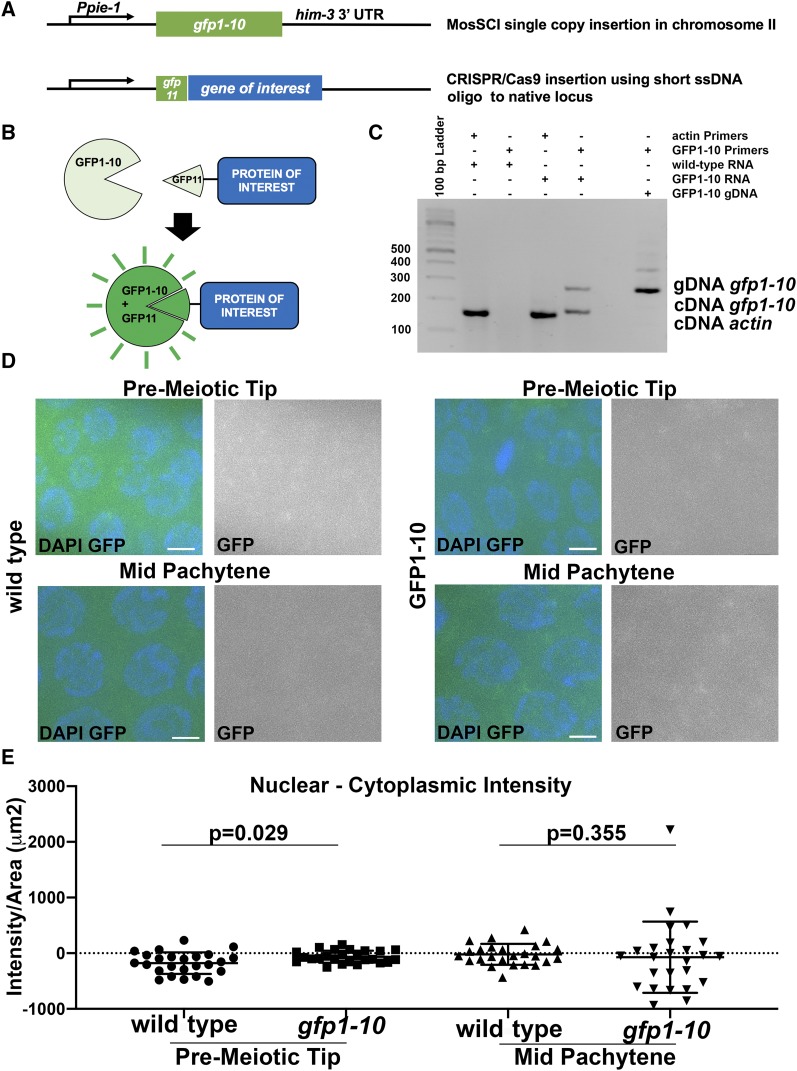
sfGFP approach in C. elegans germline A) Design of genetic loci. GFP1-10 was inserted into a locus that is permissive for germline expression on chromosome II under the control of a germline specific promoter and 3′UTR. GFP11 fragments were inserted via CRISPR to tag proteins at their endogenous location. B) Schematic representation of sfGFP approach for expression of germline proteins. C) RT-PCR on RNA extracted from *gfp1-10* worms indicates that *gfp1-10* is expressed. Actin PCR serves as a positive control to confirm RNA extraction was successful (expected RT-PCR size = 151bp). GFP1-10 RT-PCR product is expected to be 170bp, while standard PCR of genomic DNA produces a 270bp sized product. The expected product size for GFP1-10 cDNA is only observed in the GFP1-10 sample. D) Expression of GFP1-10 alone does not lead to GFP fluorescence, similar to wild-type (N2) worms. E) Quantification of fluorescence of GFP1-10 strain shows that there is no fluorescence in the absence of GFP11 tag. Nuclear intensity calculated as Intensity/Area(µm2), for pre-meiotic and mid-pachytene regions. Scale bars 2μm.

The split sfGFP system requires the large GFP1-10 subunit to be constitutively expressed in the tissue of interest, while the short *gfp11* fragment can be inserted at an endogenous locus to tag the protein of interest (Figure 1A and B). To express GFP1-10 in the *C. elegans* germline we utilized the MosSCI system. We cloned a GFP1-10 sequence (Kamiyama *et al.* 2016) that was modified for *C. elegans* codon usage (Table 1) and contained two introns. This fragment was expressed from the *pie-1* promoter and regulated by the *him-3* 3′UTR for transcription and translational regulation (Figure 1A), largely based on a previous publication demonstrating germline-specific expression of histone H2B using a similar design (Merritt *et al.* 2008). We chose the MosSCI ttTi5605 insertion site on chromosome II because it is a well-documented chromatin environment for robust germline expression (Frøkjaer-Jensen *et al.* 2008; Kaymak *et al.* 2016). The expression of GFP1-10 was confirmed by RT-PCR of whole worms (Figure 1C), but did not result in any visible fluorescence in the germline (Figure 1D). Normalized nuclear intensity measurements revealed no fluorescence specific to the nucleus (values are slightly below 0 Figure 1E). Overall levels of GFP detection were similar in wild-type untagged strain and the *gfp1-10* strain we generated, indicating that the GFP1-10 fragment does not produce a detectable GFP signal (Figure 1C and D). The insertion of *gfp1-10* had no effect on number of eggs laid per worms or the number of adult worms hatching from them (Table 2, *P* = 0.67), indicating that the expression of GFP1-10 likely has no deleterious effect. In wild-type germline six pairs of chromosomes form six bivalents at diakinesis oocytes (Lui and Colaiácovo 2012). We observed six DAPI bodies in all diakinesis oocytes of the *gfp1-10* strain (n = 23).

**Table 2:** No high incidence of males is observed in *gfp1-10* and *gfp11* tagged strains. **Table indicates the total number of adults (F1s) scored for each P0 and the number of males among these adults. These numbers are not significantly different between the strains (Fisher’s Exact test). Wild-type *C. elegans* is reported to generate 0.1% males (****Hodgkin *et al.* 1979****;** **Lui and Colaiácovo 2012****).**

Genotype	N = P0s	N = F1	N = Males	% males
wild-type	9	2038	0	0
*gfp1-10*	12	2956	4	0.13
*gfp1-10*; *gfp11*::*syp-4*	13	2953	6	0.2
*gfp1-10*; *gfp11*::*akir-1*	12	3014	1	0.03
*gfp1-10*; *gfp11*::*rpa-1*	12	2801	2	0.07
*flag*::*akir-1*	8	1393	0	0
*ollas*::*rpa-1*	7	1867	3	0.16

To validate our approach we tagged three genes at their endogenous loci with *gfp-11* to generate *gfp11*::*akir-1*, *gfp11*::*rpa-1*, and *gfp11*::*syp-4*. These three proteins were selected because the germline expression pattern of these genes is known. SYP-4 is a component of the synaptonemal complex, a meiosis-specific complex that holds homologous chromosomes together and ensures the formation of crossovers between them (Smolikov *et al.* 2009). AKIR-1 is a soma- and germline-expressed protein that is involved in synaptonemal complex disassembly (Clemons *et al.* 2013, Polanowska *et al.* 2018). RPA-1 is a subunit of the RPA complex that is involved in replication and DNA damage repair (Kamath *et al.* 2003; Martin *et al.* 2005). The GFP1-10 strain was injected with CRISPR/Cas9 protein-crRNA-tracrRNA complexes, as well as the appropriate ssODNs containing the sequence for GFP11 (Table 1). The *gfp11* insertions contained 63 bases following the first coding ATG. The *gfp11* insertions encode for 16 amino acids of GFP11, followed by a linker sequence GGGKF.

### Tagging proteins With GFP11 does not affect worm fertility or gonad structure

Addition of protein tags may impact the function of the tagged proteins. A loss of function allele of *syp-4* leads to extended transition zone morphology and univalent formation (Smolikov *et al.* 2009), *akir-1* mutants have a small germline phenotype (Clemons *et al.* 2013)], and knock-down of *rpa-1* by RNAi leads to a small germline as well (Koury *et al.* 2018). All of these phenotypes are readily observed by DAPI analysis, but all three tagged lines expressing the GFP1-10 and GFP11 tagged proteins appeared superficially wild-type with gonads that did not appear different than wild-type gonads.

**Figure 2:**
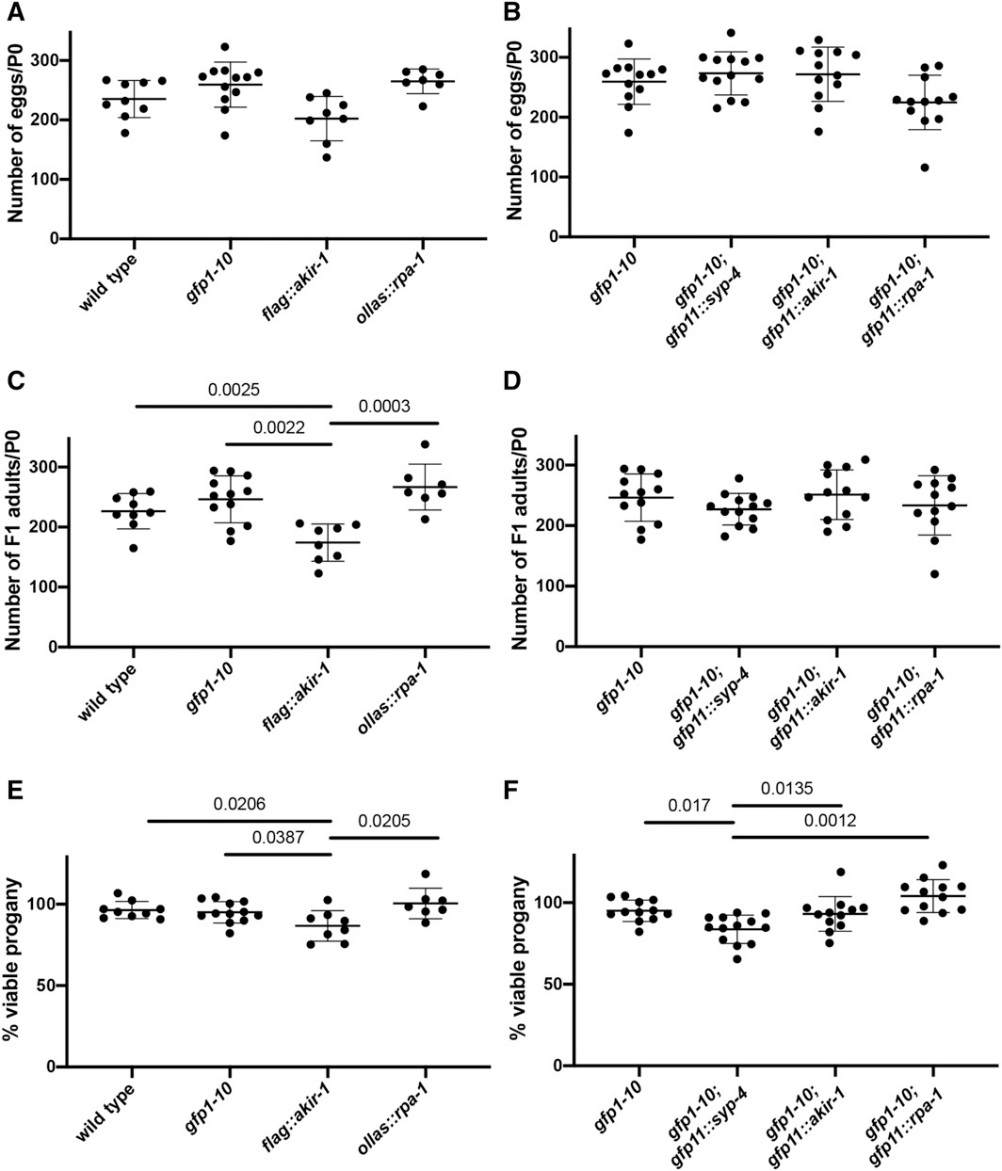
Insertion of GFP11 tag has mild or no effect on egg numbers and viability. P0 were scored for number of F1 eggs and adults. A and B) egg numbers: each data point is egg number of a single P0. C and D) F1 numbers: each data point is F1 adult number of a single P0. E and F) Viability, each data point is percent of F1 divided by egg numbers. Values that are not significant are not shown as well as 3 pairwise comparisons in which the mutant had greater viability: *ollas*::*rpa-1* had more F1s/P0 compared to wild-type (*P* = 0.0447), *gfp1-10*; *gfp11*::*rpa-1* had higher viability compared to *gfp1-10* and *gfp1-10*; *gfp11*::*rpa-1* (*P* = 0.0173). Statistics: Kruskal-Wallis test was performed on each panel (egg, adult and viability) and when significant Mann-Whitney *U*-test was performed on every pairwise combination to identify which combinations show reduced viability compared to wild-type (A, C and E) and *gfp1-10* strain (B, D and F).

To exclude that there are small effects on fertility that are not observable by DAPI analysis, we measured the effect of GFP11 tag insertion on egg viability (embryonic lethality/viability) and X-chromosome nondisjunction [high incidence of males (HIM) phenotype]. We scored the number of eggs and F1 progeny of worms (P0s) in a four-day egg lay period following the L4 developmental stage. For every genotype tested we calculated the average number of F1 eggs, adult hermaphrodites and adult males per each P0 worm (Figure 2 and Table 2). From these two values we calculated the percent of viable progeny and percent males (Figure 2 and Table 2). In wild-type worms random nondisjunction of the X chromosome occurs in low frequency leading to the generation of <0.3% males among the progeny (Hodgkin *et al.* 1979; Lui and Colaiácovo 2012). For all genotypes tested the percent of males was not significantly different from this expected value or from the percent males found in wild-type or *gfp1-10* strain in our experiment. This indicates that none of the strains involved in our study show increased X-chromosome nondisjunction.

*gfp11*::*syp-4* insertion in *gfp1-10* strain had no statistically significant effect on egg or adult progeny numbers (Figure 2 B, C and D). However, the number of eggs was slightly elevated and the number of adult progeny was slightly reduced in *gfp1-10*; *gfp11*::*syp-4*, so the combination of these two small changes lead to statistically different decrease in percent viable progeny (compared to other strains carrying *gfp1-10*, Figure 2 B, C and D). This reduction was relatively mild (from 95% in *gfp1-10* to 84% *gfp1-10*; *gfp11*::*syp-4*), indicating that *gfp1-10*; *gfp11*::*syp-4* is mostly functional. For comparison, a loss of function allele of *syp-4* leads to reduction in progeny numbers to almost none- only 2.5% of eggs are viable (Smolikov *et al.* 2009). The lack of HIM phenotype (Table 2) suggest that the X- chromosome forms chaismata. To test if chismata numbers are reduced in *gfp1-10*; *gfp11*::*syp-4* germline we counted the number of DAPI bodies in diakinesis -1 oocytes. While wild-type germline and *gfp1-10* germlines show six DAPI bodies at diakinesis oocytes, *syp-4* null mutants lack chiasma and show 11.9 DAPI bodies [this study and (Smolikov *et al.* 2009)]. We observed six DAPI bodies in all but one oocyte of *gfp1-10*; *gfp11*::*syp-4* (n = 22), resulting in 6.04+/−0.21 DAPI bodies. Although this is not statistically significant (*P* = 0.4889, Fisher’s exact test), it is possible that crossovers are not formed for one homologous chromosome pair in less than 5% of oocytes. We conclude that the *gfp1-10*; *gfp11*::*syp-4* strain contains mostly functional SYP-4 protein.

AKIR-1 is not completely essential for fertility, but *akir-1* mutants have low number of progeny due to a combination of embryonic lethality and reduction in the number of eggs laid [21% embryonic lethality, 46 ± 22 eggs/worm (Clemons *et al.* 2013)]. The number of progeny per worm in *gfp1-10*; *gfp11*::*akir-1* was indistinguishable from that of the strain carrying *gfp1-10* (Figure 2 B, C and D), supporting it being a functional protein tag. We compared the percent of viable eggs in *gfp1-10*; *gfp11*::*akir-1* to a FLAG tag strain (Bowman *et al.* 2019). Surprisingly, *flag*::*akir-1* had a mild (∼10%) effect on hatching rates due to reduction in F1 adult progeny (Figure 2 A, C and E). It is important to note that most of the contribution to the reduction in brood size in *akir-1* null comes from the reduction in number of eggs laid [46 ± 22 eggs per worm in *akir-1(rj1)*, compared to 256 ± 45 in wild-type]. However, both *akir-1* tagged lines did not show significant reduction in the numbers of eggs laid (Figure 2). Thus, based on these measurements insertion of small tags (in this case, 3XFLAG,) can lead to mild perturbation of AKIR-1 activity, while *gfp11*::*akir-1* tag results in a functional protein.

A deletion allele for *rpa-1* is not available, but based on the conserved role of RPA-1 in DNA replication and repair and the severe phenotype of *rpa-1(RNAi)*, it is likely required for viability (Koury *et al.* 2018). Like what we found for *gfp1-10*; *gfp11*::*akir-1*, the number of eggs, progeny and viability in *gfp1-10*; *gfp11*::*rpa-1* was not significantly reduced compared to a strain carrying *gfp1-10*, supporting it being a functional tag (Figure 2 B, C and D). We also compared the hatching rate between *ollas*::*rpa-1* line we generated via CRISPR [OLLAS tag: (Park *et al.* 2008)] and *gfp1-10*; *gfp11*::*rpa-1*. These two genotypes were indistinguishable from each other and from their respective controls (Figure 2). We conclude that insertion of GFP-11 creates a functional tagged RPA-1 in a way that is comparable to insertion of a small epitope tag.

### Proteins tagged With GFP11 exhibit localization patterns similar to those obtained by other methods

All three proteins tagged exhibited a distinct localization pattern in the germline. To determine if the localization pattern of split-sfGFP tagged proteins matched the localization of endogenous protein, we compared sfGFP fluorescence to immunofluorescent staining. For GFP11 tagged strains, dissected gonads were fixed in conditions that preserved GFP protein fluorescence, without the need for antibody staining (Figure 2). For SYP-4, we used an antibody generated for this protein. For AKIR-1 we used two published strains: a 3XFLAG tagged strain along with an anti-FLAG antibody (Bowman *et al.* 2019) and a *akir-1*::*gfp* strain containing AKIR-1 fused to full-length GFP, fixing as performed with *gfp11*::*akir-1* (Polanowska *et al.* 2018). RPA-1 localization was published previously, but this antibody is no longer available to visualize RPA-1 (Martin *et al.* 2005). Integrated extra chromosomal array for GFP::RPA-1 is available, but the foci are very dim, likely due to silencing that is frequently observed with multi-copy arrays (Sonneville *et al.* 2012). To obtain RPA-1 localization that is indicative of its endogenous localization, we used the *ollas*::*rpa-1* strain that we analyzed above (for viability, Table 2) and visualized RPA-1 via antibody that targets OLLAS.

**Figure 3:**
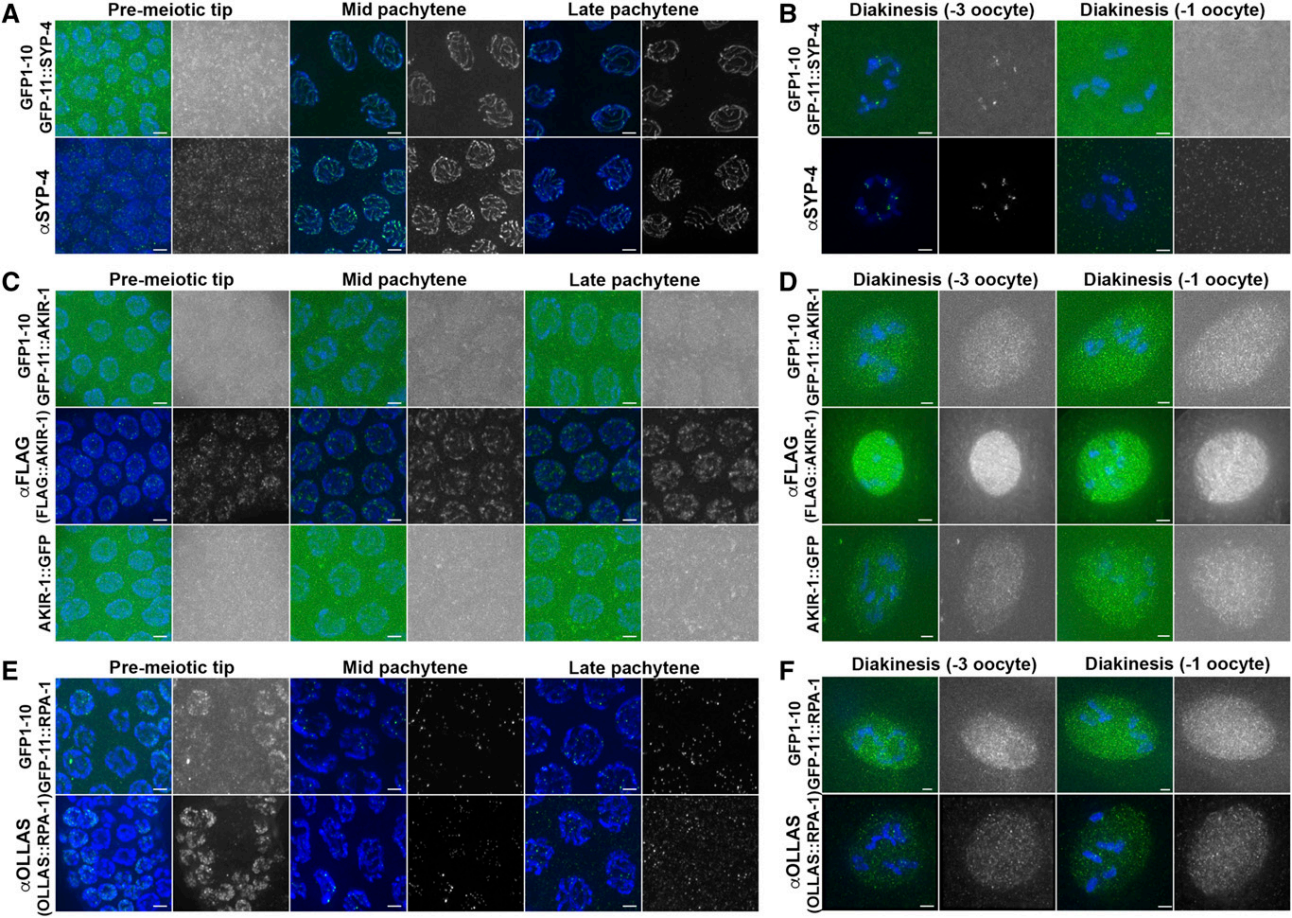
GFP11 tagged proteins are expressed in localization patterns similar to what is found by antibody staining A-B) Expression of GFP11 tagged SYP-4 in the germline expressing GFP1-10 strain leads to localization pattern essentially identical to that obtained by antibody staining for SYP-4. Green- top GFP11::SYP-4, bottom anti-SYP-4 antibody. Blue- DAPI. C-D) Expression of GFP11 tagged AKIR-1 in the germline expressing GFP1-10 strain leads to localization pattern almost identical to that obtained by AKIR-1::GFP (GFP full-length) fusion and similar to antibody staining for anti-FLAG of 3XFLAG::AKIR-1 tagged strain in diakinesis. Green- top GFP11::AKIR, bottom anti-FLAG antibody. Blue- DAPI. E-F) Expression of GFP11 tagged RPA-1 in the germline expressing GFP1-10 strain leads to localization pattern essentially identical to that obtained by antibody staining for anti-OLLAS of OLLAS tagged RPA-1 strain. Green- top GFP11::RPA-1, bottom anti OLLAS antibody. Blue- DAPI. Stages indicated above include the mitotic pre-meiotic tip, and the meiotic stages of mid pachytene, late pachytene, diakinesis (-3 oocyte) and diakinesis (-1 oocyte), when the -3 and -1 refers to their position relatively to the spermatheca (-1 being closer than -3). Greyscale images are green channel of the image to its left. Scale bars 2μm.

When compared with their antibody stained counterparts, GFP11 localization matched or was similar to the localization of the endogenous protein (Figure 3). GFP11::SYP-4 was found as a nuclear haze in mitotic nuclei (in the pre-meiotic tip) and co-localized with DAPI upon meiotic entry. Using antibody for SYP-4 we observed a linear pattern of localization between homologous chromosomes in pachytene, which is expected of a synaptonemal complex protein [Figure 2A and (Smolikov *et al.* 2009)]. In diakinesis, GFP11::SYP-4 was observed at the short arm of the bivalent and disappeared at the last oocyte prior to fertilization (Figure 2B). This localization mimicked the staining pattern in wild-type worms for the antibody specific for SYP-4.

The fluorescence of GFP11::AKIR-1 was observed dimly in all nuclei of the germline. GFP-11::AKIR-1 fluorescence was dim in the distal germline and brighter in diakinesis nuclei (Figure 3C and D). This localization pattern was almost identical to that observed with the *akir-1*::*gfp* strain (Polanowska *et al.* 2018). Using anti-FLAG antibodies in 3XFLAG::AKIR-1 gonads we observed a similar localization pattern to that of both GFP tagged strains in diakinesis [Figure 3D (Bowman *et al.* 2019)]. However, localization of 3XFLAG::AKIR-1, was overall brighter than that of GFP11::AKIR-1 or AKIR-1::GFP. A punctate pattern was observed around chromatin in the distal germline of 3XFLAG::AKIR-1, as opposed to diffused and very weak staining in GFP tagged lines. Since we observed a very similar localization pattern when AKIR-1 was tagged with GFP full-length or split-GFP, we conclude that GFP11 may have a different localization pattern compared to epitope tagging, but this localization is consisted with full-length GFP localization.

GFP11::RPA-1 formed a nuclear haze in the pre-meiotic tip, and a nuclear haze at the end of pachytene and throughout diakinesis, just as we observed with OLLAS tagged RPA-1 using anti-OLLAS antibody staining (Figure 3C and D). GFP11::RPA-1 also formed foci in pachytene, matching its OLLAS tagged counterpart and previous reports using an antibody against RPA-1 [Figure 2C and (Martin *et al.* 2005)].

### Proteins tagged With GFP11 can be observed by live imaging

**Figure 4:**
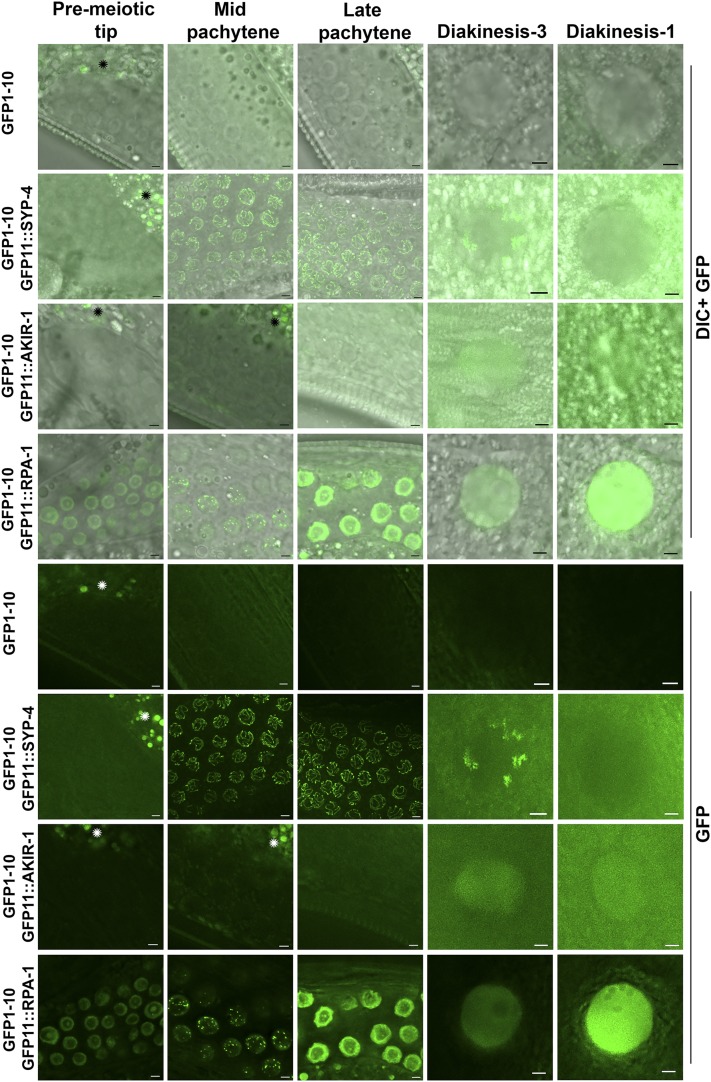
The split sfGFP approach is suitable for live imaging Expression of GFP11 tagged proteins in the germline expressing GFP1-10 strain leads to localization pattern visible by live imaging. Top is DIC channel and GFP channel, bottom are images taken from the same region just with GFP. Green- live imaging GFP channel. Stages indicated above include the mitotic pre-meiotic tip, and the meiotic stages of mid pachytene, late pachytene, diakinesis (-3 oocyte) and diakinesis (-1 oocyte). Regions marked with * are gut regions that show autofluorescence. All images are slices of 0.8μm close to the mid-section of the nuclei, except SYP-4 which is projection throughout the nuclei. Scale bars 2μm.

**Figure 5:**
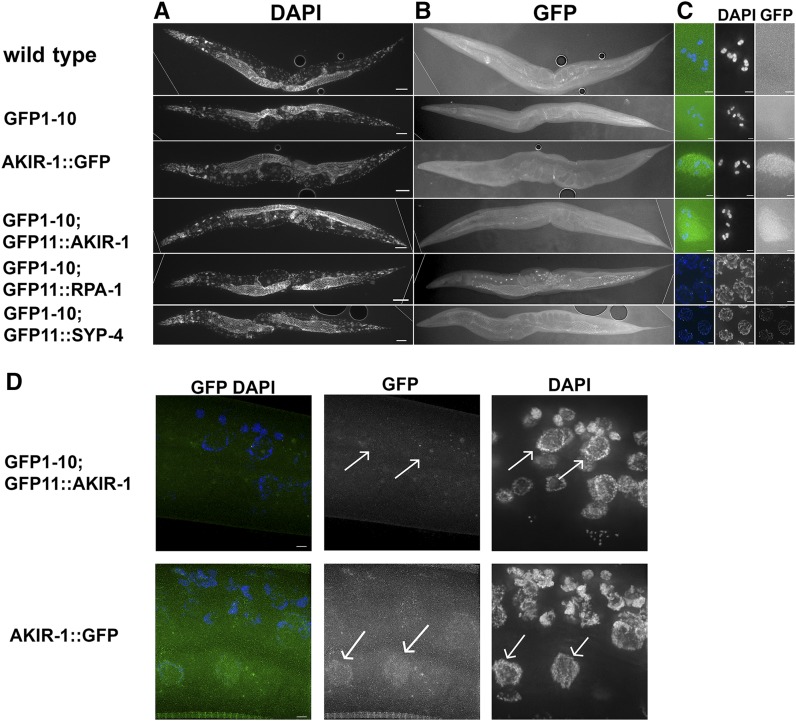
sfGFP1-10 and GFP11 tagged protein fluorescence is not observed in somatic tissue Images of ethanol fixed worms were taken for both A) DAPI and B) FITC channels. C) Zoom-in images appear to the right of the corresponding worms, and demonstrate the lack of fluorescence in diakinesis (-1 oocyte) nuclei for wild-type and GFP1-10 worms. Representative GFP fluorescence images show diakinesis (-1 oocyte) nuclei for *gfp1-10*; *gfp11*::*akir-1* and *akir-1*::*gfp* strains, mid-pachytene nuclei for *gfp1-10*; *gfp11*::*rpa-1*, and late pachytene nuclei for *gfp1-10*; *gfp-11*::*syp-4*. For all genes tested, fluorescence is not observed in somatic tissue. Dotted lines are where black background was added (externally to that line so a rectangular shape can be made). D) In L3 worm, *akir-1*::*gfp* that is expressed from the AKIR-1 promoter and regulated by its 3′UTR localizes to somatic epidermal nuclei, but the same cells in *gfp1-10*; *gfp11*::*akir-1* do not show GFP fluorescence in these nuclei (representative nuclei are marked by arrows). Scale bars in A and B is 50 µm, in C and D is 2 µm.

When live worms were examined using fluorescence microscopy, GFP expression in all three lines (*gfp11*::*akir-1*, *gfp11*::*rpa-1*, and *gfp11*::*syp-4*) was observed. (Figure 4). In *gfp-11*::*syp-4* worms, GFP was not observed in mitotic nuclei (found in the pre-meiotic tip), but instead localized to chromosomes at meiotic entry and observed in pachytene as a linear localization pattern, which is expected of a synaptonemal complex protein. GFP was observed as six patches in the diakinesis nuclei of these oocytes, a localization pattern typical of synaptonemal complex protein at this stage (short arm of the bivalent, based on fixed-sample preparation, described above). *gfp-11*::*akir-1* worms exhibited diffuse nuclear-specific fluorescence, which was only observed in diakinesis-stage nuclei. Thus, the distal nuclear localization of AKIR-1 observed by immunofluorescence staining was not detected by live imaging. In *gfp11*::*rpa-1* worms, GFP was observed in nuclei throughout the germline. RPA-1 foci were observed in the pachytene stage of meiotic prophase I germline as previously reported (Martin *et al.* 2005). Importantly, none of the GFP11 tagged proteins, including GFP1-10 parent strain were observed in somatic tissues (Figure 5). SYP-4 is a protein specifically expressed in the germline, but RPA-1 is expected to be present in each replicating cell, yet it was not observed in any somatic tissue. AKIR-1::GFP single copy insertion was shown to localize to somatic nuclei at the L3 stage but not in the adult (Polanowska *et al.* 2018). Indeed, when AKIR-1::GFP expression was derived from its endogenous promoter and UTR it was expressed in L3 somatic nuclei (Figure 5D). However, GFP11::AKIR-1 was not found in any somatic cells, including cells in the L3 stage, as expected (since GFP1-10 is regulated by germline specific elements).

**Figure 6:**
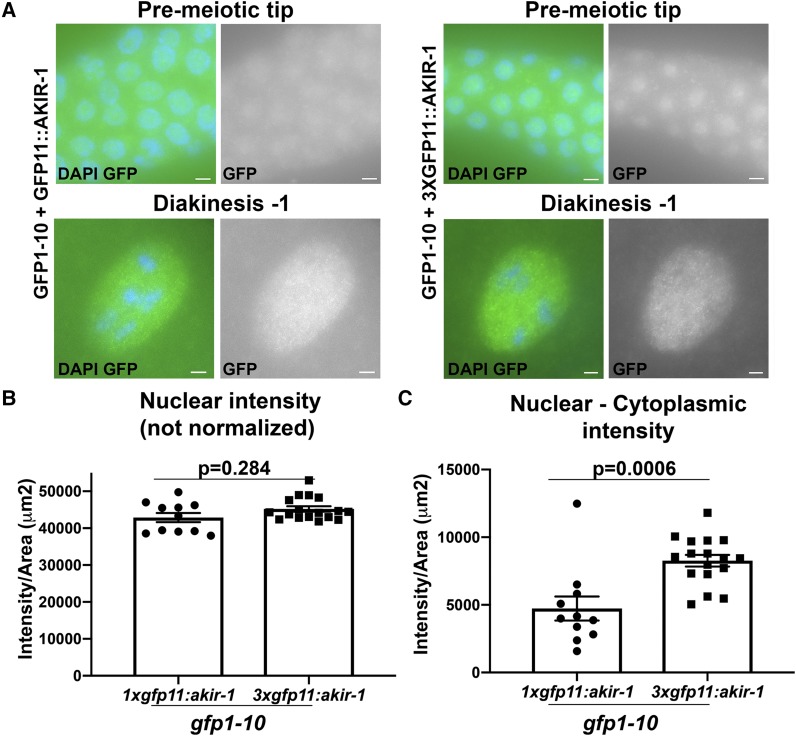
Increasing the GFP11 tag number to three slightly improves fluorescence intensity of GFP11-AKIR-1 A) Expression of 3XGFP11 tagged AKIR-1 in the germline expressing GFP1-10. Blue- DAPI, Green- GFP. Stages indicated above include the mitotic pre-meiotic tip, and the meiotic stages of diakinesis (-1 oocyte). Scale bars 2μm. B) Nuclear intensity calculated as Raw intensity/Area(µm2), for diakinesis (-1 oocyte). C) Nuclear intensity normalized: nuclear Raw intensity/Area(µm^2^) from which cytoplasmic background [Raw intensity/Area(µm^2^)] was subtracted.

Tagging a protein with tandem GFP11 repeats has been reported to increase the GFP signal in mammalian tissue culture studies (Kamiyama *et al.* 2016). This presumably occurs by recruitment of multiple GFP1-10 subunits to a single target protein marked by the tandem GFP11 array. To test if this is also occurs in our system we converted 1X*gfp11* to 3X*gfp11* using CRISPR/Cas9 at the *akir-1* and *syp-4* loci. Attempts at tagging SYP-4 with 3X*gfp11* led to loss of function of 3XGFP11::SYP-4 (12.5+/−1.9 DAPI bodies instead of 6). 3X*gfp11*::*akir-1* worms were fertile and homozygous viable and exhibited similar localization pattern to that of 1X*gfp11*::*akir-1* (Figure 6A). *gfp11*::*akir-1* exhibited relatively weak GFP fluorescence in live-imaging experiments and, therefore, could potentially be improved. When not normalized, signal intensity of 1XGFP11::AKIR-1 in diakinesis nuclei was not statistically different than that of 3XGFP11::AKIR-1 (Figure 6B). When intensity was normalized by the subtraction of cytoplasmic intensity, 3XGFP11::AKIR-1 showed increased nuclear intensity compared to 1XGFP11::AKIR-1 (Figure 6C, *P* = 0.0006), indicating that this approach can modestly increase GFP intensity for this protein.

## Discussion

Using MosSCI and CRISPR/Cas9 technology we were able to tag and observe GFP fluorescence for three proteins with no severe disruption of their function. Furthermore, we were able to restrict GFP expression to the *C. elegans* germline, allowing us to visualize their localization in live worms without interference from their expression in somatic tissues. While RPA-1 and SYP-4 expression is likely restricted to the germline in adult worms, AKIR-1 was shown to be expressed in somatic tissues of larva stage 3 worms in addition to the germline (Polanowska *et al.* 2018). In our system we did not observe any GFP11::AKIR-1 fluorescence in somatic tissue, including epidermal cells of larva stage 3 worms, as expected by our design (using germline-specific regulatory elements for GFP1-10). This, therefore, validates our hypothesis that the split sfGFP system can be used for tissue-specific tagging of proteins in *C. elegans*.

Previous reports in *C. elegans* used the split GFP technique to label ribosomes found in specific tissues [Ribosome Imaging Based On split GFP (RIBOS), (Noma *et al.* 2017)], identify interactions across synapses [GFP Reconstitution Across Synaptic Partners (GRASP) (Feinberg *et al.* 2008), and the combinatorial marking of cells (Zhang *et al.* 2004). In GRASP, two separate proteins were tagged with either the GFP1-10 or the GFP11 subunit but expressed in two different cells. When these proteins were expressed on opposite parts of the same synapse, fluorescence was observed (Feinberg *et al.* 2008). Thus, this approach was used to identify protein-protein interaction and not localization of a single protein as in our experiments. RIBOS is used for tagging a single protein, which is a similar idea to what we implemented here. In RIBOS, a ribosomal protein was tagged with the GFP11, while the GFP1-10 subunit was constitutively expressed in the epidermis. The tagged ribosomal protein was expressed from an exogenous locus and required that the endogenous gene be knocked-out. Moreover, this system required overexpression of GFP1-10 from a multicopy array (Noma *et al.* 2017). In our system GFP1-10 is not over expressed (a single copy insertion) and the GFP11 fusion protein is expressed from its endogenous locus. This has the advantage of recapitulating the *in vivo* expression levels, which is less likely to create a loss of function or gain of function phenotype. Our approach therefore bypassed the limitation of the RIBOS system that requires GFP1-10 overexpression or deletion of an endogenous locus.

We have shown that while tagging AKIR-1 or RPA-1 had no effect on viability, tagging of SYP-4 slightly reduced hatching rates, which may be due to mild defects in crossover formation. The fact that protein tagging may interfere with protein function is not unique to our system and is also true for any other tags, including small epitope tags [for example: (Goel *et al.* 2000; Bucher *et al.* 2002; Carson *et al.* 2007; Thielges *et al.* 2011; Saiz-Baggetto *et al.* 2017)]. Introducing a small tag that allows live imaging, like we have shown, may also have an advantage in the case of proteins that do not tolerate large tags (such as full-length GFP). Attempts at tagging SYP-4 with larger tags led to loss of functions of SYP-4: 3XGFP11 (this study) and full-length GFP (Colaiacovo P.M., personal communication) mutants show univalents at diakinesis, while tagging using 1XGFP11 is mostly functional. Epitope tags are ubiquitously used by *C. elegans* researchers, especially in recent years since CRISPR/Cas9 become a standard method for gene manipulation. However, these tags are not always validated for their effect on fertility by scoring egg hatching rates. Since any protein tagging may interfere with the function of the protein tagged, careful analysis of the CRISPR/Cas9 *gfp11* insertion lines is required (as would have been expected in the case of full-length insertion or epitope tagging) to exclude a minor deleterious effect.

It should be possible to use a variation of the split sfGFP technology with other colored fluorophores such as RFP or YFP, allowing the study of multiple proteins simultaneously (Kamiyama *et al.* 2016). Tandem GFP11 tagging may also prove advantageous, potentially enhancing fluorescent signal, making proteins more visible in live worms. It is also possible that increasing GFP11 repeat number may interfere with function of proteins that cannot tolerate large tags, but this is true as well for full-length GFP, and thus is not a disadvantage of the split-GFP system as compared to full-length GFP tagging. We have only successfully tagged with 3XGFP11 at a single locus, and it is possible that introducing higher number of tandem tags will lead to the desired effect of substantially increasing the GFP signal.

## References

[bib1] Bowman R. Balukof N. Ford T. Smolikove S., 2019 A Novel Role for α-Importins and Akirin in Establishment of Meiotic Sister Chromatid Cohesion in Caenorhabditis elegans. Genetics 211: 617–635. 10.1534/genetics.118.301458 30563860 PMC6366927

[bib2] Bucher M. H. Evdokimov A. G. Waugh D. S., 2002 Differential effects of short affinity tags on the crystallization of Pyrococcus furiosus maltodextrin-binding protein. Acta Crystallogr. D Biol. Crystallogr. 58: 392–397. 10.1107/S0907444901021187 11856823

[bib3] Cabantous S. Terwilliger T. C. Waldo G. S., 2005 Protein tagging and detection with engineered self-assembling fragments of green fluorescent protein. Nat. Biotechnol. 23: 102–107. 10.1038/nbt1044 15580262

[bib4] Carson M. Johnson D. H. McDonald H. Brouillette C. DeLucas L. J., 2007 His-tag impact on structure. Acta Crystallogr. D Biol. Crystallogr. 63: 295–301. 10.1107/S0907444906052024 17327666

[bib5] Chalfie M. Tu Y. Euskirchen G. Ward W. W. Prasher D. C., 1994 Green fluorescent protein as a marker for gene expression. Science 263: 802–805. 10.1126/science.8303295 8303295

[bib6] Clemons A. M. Brockway H. M. Yin Y. Kasinathan B. Butterfield Y. S., 2013 akirin is required for diakinesis bivalent structure and synaptonemal complex disassembly at meiotic prophase I. Mol. Biol. Cell 24: 1053–1067. 10.1091/mbc.e12-11-0841 23363597 PMC3608493

[bib7] Feinberg E. H. VanHoven M. K. Bendesky A. Wang G. Fetter R. D., 2008 GFP Reconstitution Across Synaptic Partners (GRASP) defines cell contacts and synapses in living nervous systems. Neuron 57: 353–363. 10.1016/j.neuron.2007.11.030 18255029

[bib8] Frøkjaer-Jensen C. Davis M. W. Hopkins C. E. Newman B. J. Thummel J. M., 2008 Single-copy insertion of transgenes in Caenorhabditis elegans. Nat. Genet. 40: 1375–1383. 10.1038/ng.248 18953339 PMC2749959

[bib9] Ghosh I. Hamilton A. D. Regan L., 2000 Antiparallel Leucine Zipper-Directed Protein Reassembly: Application to the Green Fluorescent Protein. J. Am. Chem. Soc. 122: 5658–5659. 10.1021/ja994421w

[bib10] Goel A. Colcher D. Koo J. S. Booth B. J. Pavlinkova G., 2000 Relative position of the hexahistidine tag effects binding properties of a tumor-associated single-chain Fv construct. Biochim. Biophys. Acta 1523: 13–20. 10.1016/S0304-4165(00)00086-6 11099853

[bib11] Hodgkin J. Horvitz H. R. Brenner S., 1979 Nondisjunction mutants of the nematode Caenorhabditis elegans. Genetics 91: 67. 17248881 10.1093/genetics/91.1.67 PMC1213932

[bib12] Kamath R. S. Fraser A. G. Dong Y. Poulin G. Durbin R., 2003 Systematic functional analysis of the Caenorhabditis elegans genome using RNAi. Nature 421: 231–237. 10.1038/nature01278 12529635

[bib13] Kamiyama D. Sekine S. Barsi-Rhyne B. Hu J. Chen B., 2016 Versatile protein tagging in cells with split fluorescent protein. Nat Comms 7: 11046 10.1038/ncomms11046 PMC4802074 26988139

[bib14] Kaymak E. Farley B. M. Hay S. A. Li C. Ho S., 2016 Efficient generation of transgenic reporter strains and analysis of expression patterns in Caenorhabditis elegans using library MosSCI. Dev. Dyn. 245: 925–936. 10.1002/dvdy.24426 27294288 PMC4981527

[bib15] Koury E. Harrell K. Smolikove S., 2018 Differential RPA-1 and RAD-51 recruitment in vivo throughout the C. elegans germline, as revealed by laser microirradiation. Nucleic Acids Res. 46: 748–764. 10.1093/nar/gkx1243 29244155 PMC5778493

[bib16] Lui D. Y. Colaiácovo M. P., 2012 Meiotic Development in Caenorhabditis elegans, pp. 133–170 in Advances in Experimental Medicine and Biology. Springer New York, New York. 10.1007/978-1-4614-4015-4_6 PMC3764601 22872477

[bib17] Martin J. S. Winkelmann N. Petalcorin M. I. R. McIlwraith M. J. Boulton S. J., 2005 RAD-51-dependent and -independent roles of a Caenorhabditis elegans BRCA2-related protein during DNA double-strand break repair. Mol. Cell. Biol. 25: 3127–3139. 10.1128/MCB.25.8.3127-3139.2005 15798199 PMC1069622

[bib18] Merritt C., Rasoloson D., Ko D., Seydoux G., 2008 3′ UTRs are the primary regulators of gene expression in the C. elegans germline. 18: 1476–1482. 10.1016/j.cub.2008.08.013 PMC2585380 18818082

[bib19] Noma K. Goncharov A. Ellisman M. H. Jin Y., 2017 Microtubule-dependent ribosome localization in C. elegans neurons. eLife 6 10.7554/eLife.26376 PMC5577916 28767038

[bib20] Paix A. Folkmann A. Goldman D. H. Kulaga H. Grzelak M. J., 2017 Precision genome editing using synthesis-dependent repair of Cas9-induced DNA breaks. Proc. Natl. Acad. Sci. USA 114: E10745–E10754. 10.1073/pnas.1711979114 29183983 PMC5740635

[bib21] Paix A., Schmidt H., Seydoux G., 2016 Cas9-assisted recombineering in C. elegans: genome editing using in vivo assembly of linear DNAs. Nucleic Acids Research. 10.1093/nar/gkw502 PMC5009740 27257074

[bib22] Paix A., Wang Y., Smith H. E., Lee C. Y. S., Calidas D., Lu T., Smith J., Schmidt H., Krause M. W., Seydoux G., 2014 Scalable and Versatile Genome Editing Using Linear DNAs with Micro-Homology to Cas9 Sites in Caenorhabditis elegans. Genetics. 10.1534/genetics.114.170423 PMC4256755 25249454

[bib23] Park S. H. Cheong C. Idoyaga J. Kim J. Y. Choi J.-H., 2008 Generation and application of new rat monoclonal antibodies against synthetic FLAG and OLLAS tags for improved immunodetection. J. Immunol. Methods 331: 27–38. 10.1016/j.jim.2007.10.012 18054954 PMC2864634

[bib24] Polanowska J. Chen J.-X. Soulé J. Omi S. Belougne J., 2018 Evolutionary plasticity in the innate immune function of Akirin. PLoS Genet. 14: e1007494 10.1371/journal.pgen.1007494 30036395 PMC6072134

[bib25] Saiz-Baggetto S. Méndez E. Quilis I. Igual J. C. Bañó M. C., 2017 Chimeric proteins tagged with specific 3xHA cassettes may present instability and functional problems. PLoS One 12: e0183067 10.1371/journal.pone.0183067 28800621 PMC5553802

[bib26] Smolikov S. Schild-Prufert K. Colaiacovo M. P., 2009 A yeast two-hybrid screen for SYP-3 interactors identifies SYP-4, a component required for synaptonemal complex assembly and chiasma formation in Caenorhabditis elegans meiosis. PLoS Genet. 5: e1000669 10.1371/journal.pgen.1000669 19798442 PMC2742731

[bib31] Snapp E. 2005 Design and Use of Fluorescent Fusion Proteins in Cell Biology. Current Protocols in Cell Biology, 27: 21.4.1–21.4.13. 10.1002/0471143030.cb2104s27 18228466 PMC2875081

[bib27] Sonneville R. Querenet M. Craig A. Gartner A. Blow J. J., 2012 The dynamics of replication licensing in live Caenorhabditis elegans embryos. J. Cell Biol. 196: 233–246. 10.1083/jcb.201110080 22249291 PMC3265957

[bib28] Thielges M. C. Chung J. K. Axup J. Y. Fayer M. D., 2011 Influence of histidine tag attachment on picosecond protein dynamics. Biochemistry 50: 5799–5805. 10.1021/bi2003923 21619030 PMC3133630

[bib30] Wiedenmann J. Oswald F. Nienhaus G. U., 2009 Fluorescent proteins for live cell imaging: Opportunities, limitations, and challenges. IUBMB Life, 61: 1029–1042. 10.1002/iub.256 19859977

[bib29] Zhang S. Ma C. Chalfie M., 2004 Combinatorial marking of cells and organelles with reconstituted fluorescent proteins. Cell 119: 137–144. 10.1016/j.cell.2004.09.012 15454087

